# Harmine Ameliorates Cognitive Impairment by Inhibiting NLRP3 Inflammasome Activation and Enhancing the BDNF/TrkB Signaling Pathway in STZ-Induced Diabetic Rats

**DOI:** 10.3389/fphar.2020.00535

**Published:** 2020-05-01

**Authors:** Peifang Liu, Hui Li, Yueqiu Wang, Xiaolin Su, Yang Li, Meiling Yan, Lan Ma, Hui Che

**Affiliations:** ^1^Department of Neurology, The Second Affiliated Hospital of Harbin Medical University, Harbin, China; ^2^Department of Endocrinology, The Second Affiliated Hospital of Harbin Medical University, Harbin, China; ^3^Department of Biochemistry and Molecular Biology, Indiana University School of Medicine, Indianapolis, IN, United States; ^4^The Center for Drug Research and Development, Guangdong Pharmaceutical University, Guangzhou, China; ^5^Department of Geriatrics, The Second Affiliated Hospital of Harbin Medical University, Harbin, China

**Keywords:** harmine, diabetes mellitus, cognitive dysfunction, NLRP3 inflammasome, BDNF

## Abstract

Diabetes mellitus (DM) is considered a risk factor for cognitive dysfunction. Harmine not only effectively improves the symptoms of DM but also provides neuroprotective effects in central nervous system diseases. However, whether harmine has an effect on diabetes-induced cognitive dysfunction and the underlying mechanisms remain unknown. In this study, the learning and memory abilities of rats were evaluated by the Morris water maze test. Changes in the nucleotide-binding oligomerization domain-containing protein (NOD)-like receptor family, pyrin domain containing 3 (NLRP3) inflammasome and brain-derived neurotrophic factor (BDNF)/TrkB signaling pathway were determined in both streptozotocin (STZ)-induced diabetic rats and high glucose (HG)-treated SH-SY5Y cells by western blotting and histochemistry. Herein, we found that harmine administration significantly ameliorated learning and memory impairment in diabetic rats. Further study showed that harmine inhibited NLRP3 inflammasome activation, as demonstrated by reduced NLRP3, ASC, cleaved caspase-1, IL-1β, and IL-18 levels, in the cortex of harmine-treated rats with DM. Harmine was observed to have similar beneficial effects in HG-treated neuronal cells. Moreover, we found that harmine treatment enhanced BDNF and phosphorylated TrkB levels in both the cortex of STZ-induced diabetic rats and HG-treated cells. These data indicate that harmine mitigates cognitive impairment by inhibiting NLRP3 inflammasome activation and enhancing the BDNF/TrkB signaling pathway. Thus, our findings suggest that harmine is a potential therapeutic drug for diabetes-induced cognitive dysfunction.

## Introduction

Diabetes mellitus (DM) is a chronic metabolic disease and a global epidemic (Sun et al., 2020). In 2019, the number of people suffering from DM worldwide was estimated to be 463 million, and this number is predicted to rise to 700 million in 2045 (International Diabetes Federation, https://www.diabetesatlas.org/). Epidemiological surveys have revealed that DM patients are at a higher risk to develop dementia (Morris et al., 2014; Zhang et al., 2018; Chornenkyy et al., 2019). Clinical studies have also shown that dementia and DM share many pathological features, such as cerebral atrophy and neurodegeneration (Korf et al., 2007; Kumar et al., 2008). Hyperglycemia, impaired insulin signaling, vascular dysfunction, and inflammation are considered to be contributors to diabetic-induced neurodegeneration and cognitive dysfunction (Sonnen et al., 2009; Kimura, 2016; Duarte et al., 2020).

Recent studies have clearly shown that the inflammasome is involved in the pathogenesis of central nervous system diseases by triggering interleukin (IL)-1β and IL-18 maturation (Ahmed et al., 2017; White et al., 2017; Du et al., 2018). The nucleotide-binding oligomerization domain-containing protein (NOD)-like receptor family, pyrin domain containing 3 (NLRP3) inflammasome is a well-known inflammasome that is associated with multiple neurodegenerative diseases. However, inactivation of the NLRP3 inflammasome can ameliorate these diseases (Lu et al., 2016; Sarkar et al., 2017; La Rosa et al., 2019; Swanson et al., 2019; Fox et al., 2020). DM-related studies have also revealed that NLRP3 inflammasome activation is involved in several diabetic complications, such as diabetic cardiomyopathy, diabetic nephropathy, and diabetic retinopathy (Yu et al., 2020). NLRP3 inflammasome activation is observed in the brains of db/db mice, a diabetic model that exhibits cognitive dysfunction (Zhai et al., 2018). Moreover, high glucose (HG) has been found to induce NLRP3 inflammasome activation in neurons (Ward and Ergul, 2016). These findings suggest that the NLRP3 inflammasome might be associated with the progression of diabetes-induced cognitive dysfunction.

Harmine is an analogous β-carboline alkaloid compound and possesses various biological functions. For example, harmine has been found to influence β-cell proliferation and induce adipocyte thermogenesis in the progression of DM (Dirice et al., 2016; Nie et al., 2016). Harmine can treat multiple cancers by increasing cell apoptosis, inhibiting CDKs and suppressing the AKT/mTOR signaling pathway (Song et al., 2004; Ding et al., 2019; Wu et al., 2019). Moreover, harmine also has a variety of effects, including antioxidative, anti-inﬂammatory, anti-hypertensive, and antidepressant effects, in the central nervous system (Li et al., 2018). Previous studies have revealed that harmine can ameliorate learning and memory impairment in scopolamine-induced animals (He et al., 2015; Li et al., 2018). However, whether harmine can improve cognitive dysfunction in diabetic rats is still unclear.

The aim of the present study was to evaluate the possible effect of harmine on the cognitive dysfunction induced by DM and to explore the underlying molecular mechanisms. Our *in vivo* and *in vitro* experiments provided strong evidence that harmine is a neuroprotective agent that acts by inhibiting NLRP3 inflammasome activation and enhancing BDNF/TrkB signaling pathway.

## Materials and Methods

### Model of Diabetes Mellitus and Pharmacotherapy

Male Sprague-Dawley rats (weight, 180–220 g) were obtained from the Animal Center of the Second Affiliated Hospital of Harbin Medical University (China). The rats were housed in a temperature (23 ± 1°C)- and humidity (55 ± 5%)-controlled environment with free access to food and water. A model of diabetes mellitus (DM) was established as described in our previous studies (Meng et al., 2019; Che et al., 2020). Briefly, the rats received a single intraperitoneal injection of 60 mg/kg streptozotocin (STZ) dissolved in citrate buffer (pH = 4.5). Fasting blood glucose (FBG) levels were detected 3 days after STZ injection. Rats with FBG levels >16.7 mmol/L were considered diabetic. Diabetic model rats were randomly divided into the DM group and the DM plus harmine treatment (DM + har) group. Beginning on day 4 after STZ injection, the rats in the DM + har group (n = 8) were given harmine (20 mg/kg) by oral gavage for 12 weeks. The rats in the DM group (n = 8) and nondiabetic (ND) group (n = 8) were orally administered an equal volume of 0.9% saline solution daily.

### Morris Water Maze

To determine the effect of harmine on cognitive function in diabetic rats, we subjected the rats to the Morris water maze test after 12 weeks of intervention. Briefly, the escape platform was placed in the first quadrant (2 cm under the surface of the water). On the first day, each rat was placed into the water facing the pool wall and then allowed to find the escape platform within 120 s by itself. If the rat failed to find the target within a specified time, one of the experimenters guided it to the platform and allowed it to rest for at least 20 s. Training was performed for 5 days. The probe trial, in which the escape platform was removed from the first quadrant, and each rat underwent a 120 s swim trial, was performed on the sixth day. The swim distance, escape latency, number of platform crossings in the target quadrant, and time spent in the target quadrant were recorded by the DigBehav-Morris Water Maze Video Analysis System.

### Cell Culture and Treatment

Human SH-SY5Y neuroblastoma cells were cultured at a density of 1×10^6^ cells/well in 6-well plates with Dulbecco's modified Eagle's medium containing 10% fetal bovine serum (FBS), 100 units/ml penicillin, and 100 μg/ml streptomycin. The cells were incubated in a common incubator with 5% CO_2_ and 95% O_2_ at 37°C. The medium was replaced every two days. When the SH-SY5Y cells were approximately 70%–80% confluent, they were exposed to HG (33 mM) conditions and treated with or without harmine (1 μM) for 48 h.

### Western Blot Analysis

Protein samples were extracted from rats from the different groups and SH-SY5Y cells for immunoblotting analysis. Briefly, the rats were anesthetized with 10% chloral hydrate (500 mg/kg, intraperitoneal) and then killed by cervical dislocation. The brain tissues were removed and homogenized in 1,000 μl RIPA buffer containing 10 μl protease inhibitor cocktail per 100 mg brain tissue. The homogenates were placed on ice for 30 min. After centrifugation at 13,500 rpm at 4°C for 30 min, the supernatants were collected. The cells were seeded in 6-well plates and treated with or without harmine. After being washed three times with PBS, the cells were lysed with RIPA buffer containing 1% protease inhibitor cocktail. The concentrations of the protein samples were assessed with the BCA kit according to the manufacturer's instructions. The proteins were separated on 10% SDS gels, transferred onto NC membranes, and incubated in 5% bovine serum albumin for 2 h. The membranes were incubated with primary antibodies against nucleotide-binding oligomerization domain-containing protein (NOD)-like receptor family, pyrin domain containing 3 (NLRP3) (1:500, Bioss, China), adaptor protein apoptosis-associated speck-like protein (ASC) (1:200, Abcam, USA), caspase-1 (1:200, Abcam, USA), interleukin-18 (IL-18) (1:200, Abcam, USA), IL-1β (1:200, Abcam, USA), brain-derived neurotrophic factor (BDNF) (1:500, Bioss, China), TrkB (1:500, Bioss, China), phosphorylated TrkB (p-TrkB) (1:200, Bioss, China), and GAPDH (1:1000) 4°C overnight. On the next day, the membranes were incubated with secondary antibodies at room temperature for 1 h. The bands were captured on the Odyssey Infrared Imaging System (LI-COR) and quantified with Odyssey software by measuring the band intensity (area × OD) and normalizing it to that of GAPDH.

### Immunofluorescence Analysis

The cells were fixed in 4% paraformaldehyde, permeabilized, and blocked in 10% serum with 0.01% triton for 1 h at room temperature. The samples were then incubated with primary antibodies against NLRP3 (1:200, Bioss, China) and BDNF (1:200, Bioss, China) overnight at 4°C. An undiluted Alexa Fluor^®^ 568 (red)-conjugated goat anti-rabbit IgG or Alexa Fluor^®^ 488 (green)-conjugated anti-rabbit IgG secondary antibody was used. Images were captured with a Zeiss microscope and quantified with Image Pro Plus 6 software.

### Immunohistochemical Staining

Rats from the different groups (n = 3 in each group) were anesthetized with 10% chloral hydrate (500 mg/kg, intraperitoneal), and the whole body of each rat was perfused through the left ventricle with 4% paraformaldehyde (pH 7.4). The cortex and hippocampi were removed from the brain of each rat and fixed in 4% paraformaldehyde overnight at 4°C. After dehydration in a concentration gradient of ethanol solutions and dimethylbenzene, the brain tissue samples were embedded in paraffin and cut into 5-μm-thick cross sections. The specimens were then deparaffinized and blocked with bovine serum albumin. The following primary antibodies were used in this study: NLRP3 (1:200, Bioss, China), caspase-1 (1:200, Abcam, USA), IL-18 (1:200, Abcam, USA), and IL-1β (1:200, Abcam, USA). Incubation was performed overnight at 4°C. After incubation with secondary antibodies at 37°C for 30 min, the sections were stained with diaminobenzidine and hematoxylin. Images were captured by microscopy (Zeiss, Germany). Ten images per rat were randomly selected, and at least thirty images per group were analyzed to determine the ratio of positive signal/image by using Image Pro Plus 6 software.

### Statistical Analysis

The data are expressed as the mean ± SEM. One-way repeated measure analysis of variance (ANOVA) was used to analyze differences in day-to-day performance in the MWM test. Student's t-test was used to analyze the differences between two groups. All statistical analyses were performed with SPSS 22.0 software. *P* < 0.05 was considered statistically significant.

## Results

### Harmine Ameliorates Learning and Memory Impairment in STZ-Induced Diabetic Rats

**Figure 1:**
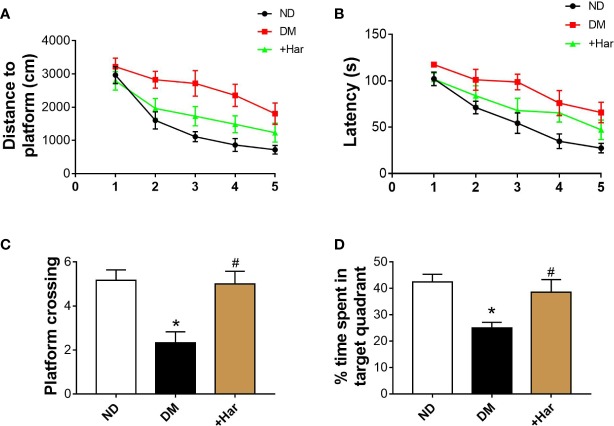
Harmine ameliorates learning and memory impairments in streptozotocin (STZ)–induced diabetic rats. **(A)** Distances to platform in the different groups on day 1 to day 5 were compared (ANOVA: Day 1: *P* = 0.535; Day 2: *P* = 0.18; Day 3: *P* = 0.005; Day 4: *P* = 0.005; Day 5: *P* = 0.031). **(B)** Latencies for the rats in the different groups to reach the platform on day 1 to day 5 were compared (ANOVA: Day 1: *P* = 0.127; Day 2: *P* = 0.133; Day 3: *P* = 0.034; Day 4: *P* = 0.041; Day 5: *P* = 0.034). **(C)** The number of platform crossings in target quadrant in the probe trial. n = 6 in each group. ^*^P < 0.05 vs sham, ^#^*P* < 0.05 vs DM. **(D)** The percentage of time spent in target quadrant in the probe trial. n = 6 rats in each group. ^*^P < 0.05 vs sham, ^#^*P* < 0.05 vs DM. DM, diabetes mellitus; Har, harmine; ND, non-diabetes.

To examine the effect of harmine on the learning and memory abilities of streptozocin (STZ)-induced diabetic rats, we subjected different groups of rats to the Morris water maze test. As illustrated in **Figure 1A**, in the training session, the diabetic rats swam a longer distance to reach the hidden platform than the non-diabetes (ND) rats. However, harmine administration significantly shortened the swimming distance of the diabetic rats. Moreover, the diabetic rats exhibited a longer escape latency than the ND rats, and this increase in escape latency was reversed by harmine administration (**Figure 1B**). In the probe trial, the number of platform crossings in the target quadrant exhibited by the diabetic rats was decreased compared with that exhibited by the ND rats. In addition, the diabetic rats spent less time in the target quadrant than the ND rats. Importantly, harmine administration increased both the number of platform crossings and the time spent in the target quadrant compared with those exhibited by the diabetic rats (**Figures 1C, D**). These data indicated that harmine can ameliorate learning and memory impairments induced by diabetes.

### Harmine Reduces NLRP3 Inflammasome Activation in STZ-Induced Diabetic Rats

**Figure 2:**
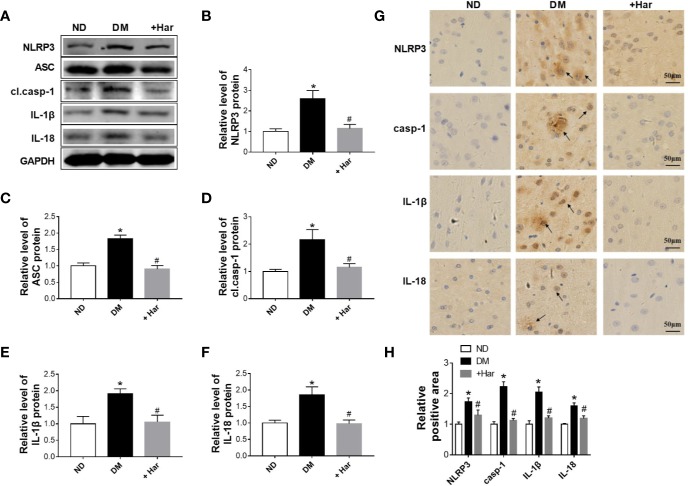
The effect of harmine on the NLRP3 inflammasome in streptozotocin (STZ)–induced diabetic rats. **(A–F)** Western blot analysis of NLRP3, ASC, cl.casp-1, IL-1β, and IL-18 levels in the cortex of STZ-induced diabetic rats after harmine administration. Western blot images **(A)** and related histograms **(B–F)**. n =3 in each group. ^*^P < 0.05 vs sham, ^#^*P* < 0.05 vs DM. **(G)** Immunohistochemical staining analysis showed that harmine reduced the expression of NLRP3, casp-1, IL-1β, and IL-18 in the cortex of diabetic rats. **(H)** Histogram of the data in G. DM, diabetes mellitus; casp-1, caspase-1; cl. casp-1, cleaved caspase-1; Har, harmine; ND, non-diabetes.

Next, we aimed to evaluate the effect of harmine on the nucleotide-binding oligomerization domain-containing protein (NOD)-like receptor family, pyrin domain containing 3 (NLRP3) inflammasome activation in STZ-induced diabetic rats. As shown in **Figures 2A, C**, the levels of major NLRP3 inflammasome components and effectors, including the NLRP3 receptor and the adaptor protein apoptosis-associated speck-like protein (ASC), were significantly increased in the brains of the diabetic rats compared with the ND rats. Moreover, NLRP3 inflammasome activation was observed in the diabetic rats, as demonstrated by higher levels of cleaved caspase-1, interleukin (IL)-1β, and IL-18. Importantly, we found that these changes were markedly reversed by harmine administration (**Figures 2A, D–F**). Moreover, immunohistochemical analysis further confirmed that the levels of the NLRP3 receptor, caspase-1, IL-1β, and IL-18 were significantly decreased by harmine treatment (**Figures 2G, H**). These results suggested that harmine can inhibit NLRP3 inflammasome activation in STZ-induced diabetic rats.

### Effect of Harmine on NLRP3 Inflammasome Activation in HG-Treated SH-SY5Y Cells

**Figure 3:**
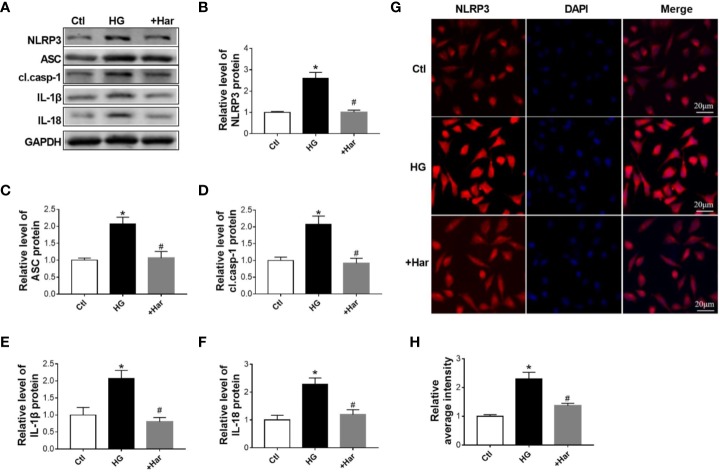
The effect of harmine on the NLRP3 inflammasome in high glucose (HG)–treated cells. **(A–F)** Western blot analysis of NLRP3, ASC, cl. casp-1, IL-1β, and IL-18 levels in HG-treated SH-SY5Y cells after harmine administration. Western blot images **(A)** and related histograms **(B–F)**. ^*^P < 0.05 vs sham, ^#^*P* < 0.05 vs HG. The data are presented as the mean ± SEM of 4 batches of cells per group. **(G)** Representative images showing staining for NLRP3 (red) and DAPI (blue) after exposure to harmine. **(H)** Histogram of the data in G. HG, high glucose; casp-1, caspase-1; cl. casp-1, cleaved caspase-1; Har, harmine.

To further observe the function of harmine on NLRP3 inflammasome activation, western blotting and immunofluorescence were used to assess changes in the NLRP3 inflammasome in high glucose (HG)-treated neuronal cells treated with or without harmine. As shown in **Figures 3A–C**, the expression of NLRP3 and ASC was found to be markedly higher in the HG-treated cells than in the control cells, and this increase in expression was reversed by harmine treatment. Moreover, HG conditions significantly upregulated cleaved caspase-1, IL-1β, and IL-18 levels. However, this effect was blocked by harmine treatment (**Figures 3A, D–F**). A similar change in NLRP3 was also observed by immunofluorescence (**Figures 3G, H**). These results suggested that harmine can inhibit NLRP3 inflammasome activation in HG-treated neuronal cells.

### Harmine Significantly Enhances BDNF/TrkB Signaling Both *In Vivo* and *In Vitro*

**Figure 4:**
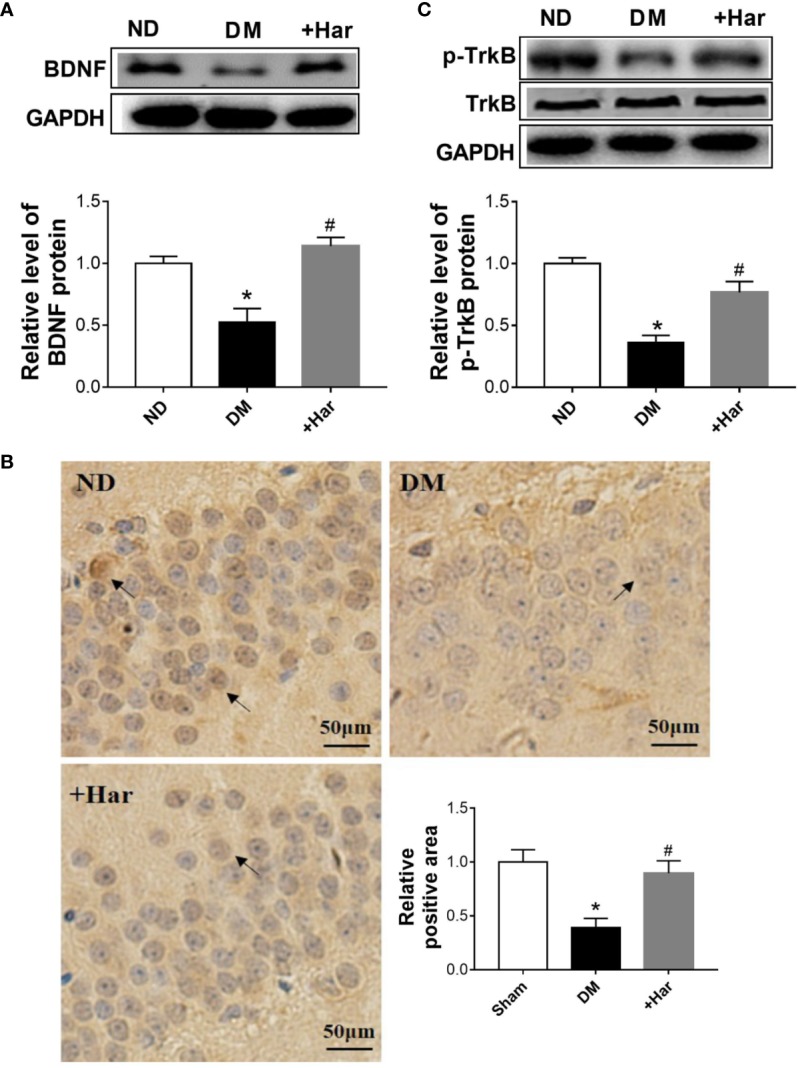
The effect of harmine on brain-derived neurotrophic factor (BDNF)/TrkB in vivo. **(A)** Harmine increased the level of BDNF in the cortex of streptozotocin (STZ)–induced diabetic rats, as detected by western blotting. Top, western blot images of BDNF. Bottom, related histogram. n = 3 in each group. *P < 0.05 vs sham, ^#^*P* < 0.05 vs DM. **(B)** Immunohistochemical analysis of BDNF in the hippocampi of diabetic rats after harmine treatment. Lower right panel, quantification of the BDNF-positive area in the brain. **(C)** Western blot analysis of p-TrkB in the cortex of STZ-induced diabetic rats after harmine treatment. Top, western blot images. Bottom, related histogram. n = 3 in each group. *P < 0.05 vs sham, ^#^*P* < 0.05 vs DM. DM, diabetes mellitus; Har, harmine; p-TrkB, phosphorylated TrkBl; ND, non-diabetes.

Previous studies have shown that BDNF/TrkB signaling is involved in the progression of diabetes-induced cognitive dysfunction. To determine the effect of harmine on BDNF/TrkB signaling, we measured the changes in BDNF and TrkB in the different groups. The results showed that STZ injection significantly reduced the level of BDNF in the brains of rats, and that this effect was reversed by harmine treatment (**Figures 4A, B**). As shown in **Figure 4C**, the level of phosphorylated TrkB (p-TrkB) was also lower in the DM group than in the ND group. However, the harmine-treated rats exhibited a higher level of p-TrkB than the DM rats.

**Figure 5:**
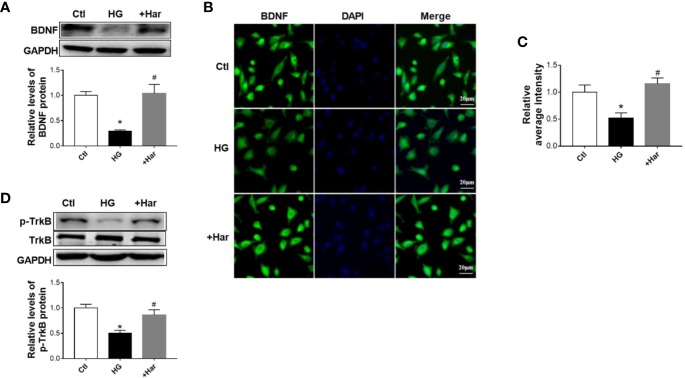
The effect of harmine on brain-derived neurotrophic factor (BDNF)/TrkB in vitro. **(A)** Harmine increased the level of BDNF in HG-treated cells, as detected by western blotting. Top, western blot images. Bottom, related histogram. n =3 in each group. *P < 0.05 vs control, ^#^*P* < 0.05 vs HG. **(B)** Immunohistochemical staining analysis showing the expression of BDNF. **(C)** Histogram of the data in B. **(D)** Western blot analysis of p-TrkB in SH-SY5Y cells exposed to HG conditions. Top, western blot images. Bottom, related histogram. n =3 in each group. *P < 0.05 vs control, ^#^*P* < 0.05 vs HG. HG, high glucose; Har, harmine; p-TrkB, phosphorylated TrkB.

In addition, we detected the effect of harmine on BDNF/TrkB signaling *in vitro*. Using western blotting, we found that the level of BDNF was much lower in the HG-treated cells than in the control cells, and was upregulated by harmine treatment (**Figure 5A**). Similar changes in BDNF were also observed by immunofluorescence (**Figures 5B, C**). Furthermore, we observed that the harmine-treated cells exhibited a higher level of p-TrkB than the HG-treated cells (**Figure 5D**). These data demonstrated that harmine can enhance the BDNF/TrkB signaling pathway both *in vivo* and *in vitro*.

## Discussion

The present study is the first to demonstrate that harmine can attenuate cognitive dysfunction in STZ-induced diabetic rats. Moreover, harmine was found to inhibit NLRP3 inflammasome activation and enhance the BDNF/TrkB signaling pathway in both STZ-induced diabetic rats and HG-treated neuronal cells.

Recently, studies have demonstrated a strong relationship between DM and dementia. Aging, hyperglycemia, insulin resistance, and inflammation are considered common pathogenic factors for DM and dementia. For example, hyperglycemia has been implicated as an important factor in neuronal cell death, synaptic plasticity dysfunction, and learning and memory impairment (Chen et al., 2019). Insulin resistance is involved in the progression of cognitive dysfunction through promoting Aβ accumulation and tau aggregation. Moreover, growing evidence suggests that inflammation plays a vital role in the relationship between DM and dementia. It has been found that the levels of inflammation-related markers, such as IL-1β, IL-18, and IL-6, are increased in both DM and dementia patients. Notably, IL-1β has been found to be associated with Aβ accumulation, tau hyperphosphorylation, β cell proliferation, and insulin secretion (Boni-Schnetzler et al., 2018; Li et al., 2020). It is widely acknowledged that NLRP3 inflammasome activation contributes to DM and dementia by triggering IL-1β maturation. NLRP3 inflammasome activation along with cognitive dysfunction has been observed in diabetic mice (Zhai et al., 2018). NLRP3 inflammasome inhibition improves cognitive dysfunction in diabetic rats following stroke (Ward et al., 2019). HG conditions can induce NLRP3 inflammasome activation in HT22 cells (Ward and Ergul, 2016). Concordant with previous findings, we found that the NLRP3 inflammasome was more strongly activated in the brains of the diabetic rats than the ND rats, as evidenced by the increased concentrations of NLRP3, ASC, caspase-1, IL-1β, and IL-18. Importantly, harmine administration significantly inhibited NLRP3 inflammasome activation and improved cognitive dysfunction in the diabetic rats. We also observed that HG conditions induced NLRP3 inflammasome activation and IL-1β and IL-18 maturation in SH-SY5Y neuronal cells, and that this effect was also blocked by harmine treatment. These results thus demonstrate that harmine exerts a neuroprotective effect by influencing the NLRP3 inflammasome both *in vivo* and *in vitro*.

The neurotrophic factor brain-derived neurotrophic factor (BDNF)/TrkB signaling pathway contributes to neuronal migration, neuronal survival, synaptic plasticity, and memory (Carabalona et al., 2016; Tomassoni-Ardori et al., 2019). Decreased levels of BDNF and TrkB are observed in rats with diabetes-induced cognitive dysfunction. However, enhancement of the BDNF/TrkB pathway alleviates cognitive impairment in diabetic rats (Yang and Gao, 2017) and protects against neuronal apoptosis and synaptic plasticity dysfunction under HG conditions (Zhong et al., 2019). Recent studies have established a link between the NLRP3 inflammasome and BDNF. IL-1β can suppress BDNF-dependent synaptic plasticity and cognitive decline through disturbing the BDNF signaling pathway (Tapia-Arancibia et al., 2008; Tong et al., 2012). Notably, NLRP3 inflammasome inhibition can upregulate BDNF expression by suppressing IL-1β (Fu et al., 2020). Consistent with previous studies, our data showed that BDNF levels were downregulated by DM, and upregulated by harmine administration. In addition, TrkB (BDNF receptor) levels were reduced in the diabetic group than in the control group. Importantly, harmine administration inhibited the NLRP3 inflammasome, significantly enhanced the BDNF/TrkB signaling pathway and ameliorated cognitive dysfunction in diabetic rats.

Our study provides evidence to show that harmine can mitigate cognitive impairment in STZ-induced diabetic rats and exert neuroprotective effects through inhibiting NLRP3 inflammasome activation and enhancing the BDNF/TrkB signaling pathway. We thus provide novel insight and potential targets for diabetes-induced cognitive dysfunction.

## Data Availability Statement

All datasets generated for this study are included in the article/supplementary material.

## Ethics Statement

All animal procedures in the current study were performed in accordance with the guidelines of the Institutional Animal Care and Use Committee of Harbin Medical University (No. SYDW 2019-123).

## Author Contributions

LM and HC were responsible for study design, analysis, and article writing. PL and MY performed the animal experiments. PL, YW, YL, and HL performed the experiments *in vitro*. LM, HC, and XS revised the manuscript.

## Funding

This work was supported by the National Natural Science Foundation of China (81600935), the China Postdoctoral Science Foundation (2017M621308), and the Natural Science Foundation of Heilongjiang Province (QC2017108), and the Major Program of Natural Science Foundation of Heilongjiang Province (ZD2018017).

## Conflict of Interest

The authors declare that the research was conducted in the absence of any commercial or financial relationships that could be construed as a potential conflict of interest.

## Abbreviations

ASC, adaptor protein apoptosis-associated speck-like protein; BDNF, brain-derived neurotrophic factor; DM, diabetes mellitus; FBG, fasting blood glucose; Har, harmine; HG, high glucose; IL-1β, Interleukin-1β; IL-18, Interleukin-18; ND, non-diabetes; NLRP3, nucleotide-binding oligomerization domain-containing protein (NOD)-like receptor family, pyrin domain containing 3; p-TrkB, phosphorylated TrkB; STZ, Streptozotin.
